# Assessing clinical cure of empirical piperacillin/tazobactam for ESBL urinary tract infections (ACCEPT—UTI)

**DOI:** 10.1093/jacamr/dlad055

**Published:** 2023-05-11

**Authors:** Sylvia S Stefanos, Sami Sakaan, Michael Samarin, Michael S Gelfand, Kerry O Cleveland, Jessie Gant, Sydney Kermeen, Diana A Hobbs, Athena L V Hobbs

**Affiliations:** Department of Pharmacy, Methodist University Hospital, 1265 Union Avenue, Memphis, TN 38104, USA; Department of Pharmacy, Methodist University Hospital, 1265 Union Avenue, Memphis, TN 38104, USA; Department of Pharmacy, Methodist University Hospital, 1265 Union Avenue, Memphis, TN 38104, USA; Infectious Diseases, Methodist University Hospital, 1265 Union Avenue, Memphis, TN 38104, USA; Division of Infectious Diseases, University of Tennessee Health Science Center, 875 Monroe Avenue, Memphis, TN 38163, USA; Infectious Diseases, Methodist University Hospital, 1265 Union Avenue, Memphis, TN 38104, USA; Division of Infectious Diseases, University of Tennessee Health Science Center, 875 Monroe Avenue, Memphis, TN 38163, USA; College of Pharmacy, University of Tennessee Health Science Center, 881 Madison Ave., Memphis, TN 38163, USA; College of Pharmacy, University of Tennessee Health Science Center, 881 Madison Ave., Memphis, TN 38163, USA; Department of Radiology, Washington University School of Medicine, 4525 Scott Ave., St Louis, MO 63110, USA; Cardinal Health Innovative Delivery Solutions, 7000 Cardinal Place, Dublin, OH 43017, USA

## Abstract

**Background:**

Data are limited regarding use of piperacillin/tazobactam for ESBL urinary tract infections (UTIs). The objective of this study was to compare clinical outcomes of patients treated empirically with piperacillin/tazobactam versus carbapenems for ESBL UTIs.

**Methods:**

This retrospective, observational, propensity score-matched study evaluated adults with an ESBL on urine culture. Patients who had UTI symptoms or leukocytosis, and who received a carbapenem or piperacillin/tazobactam empirically for at least 48 h were included. The primary outcome was clinical success within 48 h, defined as resolution of temperature (36–38°C), resolution of symptoms or leukocytosis (WBC <12 × 10^3^/μL) in the absence of documented symptoms, and the absence of readmission for an ESBL UTI within 6 months. Secondary outcomes included time to clinical resolution, hospital length of stay, and in-hospital and 30 day all-cause mortality.

**Results:**

Overall, 223 patients were included in the full cohort and 200 patients in the matched cohort (piperacillin/tazobactam = 100, carbapenem = 100). Baseline characteristics were similar between the groups. There was no difference in the primary outcome of clinical success between the carbapenem and piperacillin/tazobactam groups (58% versus 56%, respectively; *P* = 0.76). Additionally, there was no difference in median (IQR) time to clinical resolution [38.9 h (21.5, 50.9 h) versus 40.3 h (27.4, 57.5 h); *P* = 0.37], in-hospital all-cause mortality (3% versus 3%; *P* = 1.00), or 30 day all-cause mortality (4% versus 2%; *P* = 0.68) between the carbapenem and piperacillin/tazobactam groups, respectively.

**Conclusions:**

There was no significant difference in clinical success for patients treated empirically with piperacillin/tazobactam compared with carbapenems for ESBL UTIs.

## Introduction

Gram-negative bacteria that produce ESBLs continue to be a serious threat to public health. According to the antibiotic threats report released by the CDC, there were an estimated 197400 cases of ESBL-producing Enterobacterales among hospitalized patients and 9100 estimated deaths in the USA in 2017.^1^ Although exposure to healthcare settings poses the greatest risk, ESBL-producing Enterobacterales can also cause community-acquired infections in otherwise healthy individuals, often in the form of urinary tract infections (UTIs).^1^

Carbapenems are generally considered the drug class of choice for ESBL infections, but there are growing concerns about overuse leading to an increase in the development of carbapenem-resistant organisms. The MERINO trial compared the use of piperacillin/tazobactam with carbapenems for ESBL bloodstream infections and found piperacillin/tazobactam to be inferior with regards to 30 day mortality.^2^ However, post hoc analyses showed no difference when the infecting organism was shown to be susceptible to piperacillin/tazobactam.^3^ Nonetheless, providers are more inclined to use carbapenems empirically given the outcomes of this trial and the most recent guidelines on the treatment of antimicrobial-resistant Gram-negative infections, which recommend the use of carbapenem therapy over piperacillin/tazobactam for ESBL pyelonephritis or complicated UTIs.^4^ Previous observational studies evaluating the definitive use of piperacillin/tazobactam for non-bacteraemic ESBL-related UTIs have suggested no difference in clinical response compared with carbapenems.^5–8^ However, data are limited regarding clinical response with the empirical use of piperacillin/tazobactam versus carbapenems for ESBL UTIs.

Although previous literature has evaluated clinical outcomes of patients given definitive therapy with piperacillin/tazobactam versus a carbapenem, published data are lacking regarding the impact of empirical choice of antibiotic. In an era where ESBL rates are increasing and appropriate empirical therapy can impact mortality outcomes, this study evaluated whether patients who ended up having an ESBL UTI were harmed by receiving empirical piperacillin/tazobactam as opposed to a carbapenem.

## Materials and methods

### Study design

This was a multicentre, retrospective, cohort study evaluating patients admitted to five adult hospitals within the Methodist Le Bonheur Healthcare (MLH) system between 1 January 2016 through 30 June 2021. The Cerner Millennium^®^ electronic medical record (EMR) system was used to review electronic health records across the MLH system for inpatient and outpatient records, and data were managed using the REDCap^TM^ (Research Electronic Data Capture) electronic database hosted at MLH.^9^

### Patient population

Patients were identified using a report generated within Cerner Millennium^®^ of all patients who had a urine culture positive for an ESBL-producing organism and received piperacillin/tazobactam or a carbapenem. Patients were then manually screened and included if they had at least one qualifying symptom or leukocytosis (WBC >12 × 10^3^/μL) in the absence of documented symptoms and if they received either a carbapenem or piperacillin/tazobactam empirically for at least 48 h. Patients were excluded if they were <18 years of age, had a concomitant source of infection at any other site excluding bacteraemia with the same organism, had polymicrobial growth (three or more organisms) on urine culture, initiated a carbapenem or piperacillin/tazobactam ≥48 h after the time of urine culture collection, received ≥48 h of an antibiotic prior to the study drug, had an ESBL isolate resistant to the empirical agent of choice, or if they had asymptomatic bacteriuria and the absence of leukocytosis at the time of initiation of a carbapenem or piperacillin/tazobactam.

### Definitions

Patients were required to have a qualifying symptom including dysuria, urinary frequency or urgency, costovertebral angle/flank tenderness, suprapubic or abdominal pain, or hypotension (systolic blood pressure <90 mmHg or mean arterial pressure <65 mmHg). If symptoms were not documented in the EMR, leukocytosis at the time of empirical antibiotic initiation was required for inclusion. Temperature resolution was defined as a documented temperature >36°C and <38°C within 48 h of initiating piperacillin/tazobactam or a carbapenem with no further abnormality in temperature while receiving the empirical antibiotic of choice. UTI classifications included complicated cystitis, uncomplicated cystitis and pyelonephritis. Complicated cystitis was defined as patients who were pregnant, had anatomical or functional urinary tract abnormalities (e.g. nephrolithiasis confirmed with imaging, ureteric stents or nephrostomy tubes, prostatitis, renal abscess/cysts, neurogenic bladder), had concurrent immunocompromising diseases or medications (defined as those receiving cytotoxic chemotherapy, immunosuppressants including >20 mg prednisone or equivalent per day for 28 days or more, or immunomodulatory biological agents; receipt of chimeric antigen receptor T-cell therapy or haematopoietic stem cell transplant within the previous 2 years; untreated or advanced human immunodeficiency virus with CD4 <200 cells/mL), and those with sepsis or septic shock defined according to standard criteria.^10^ Patients were classified with pyelonephritis if they had fever with either costovertebral angle/flank pain or leukocytosis or if there was evidence of pyelonephritis on imaging with ultrasound or CT. Uncomplicated cystitis was classified as patients who did not meet any criteria for complicated cystitis or pyelonephritis. Patients were also classified with a catheter-related UTI in addition to a previous classification if they presented with a chronic catheter or developed a UTI after 48 h of a temporary indwelling catheter. Patients were considered to have a history of recurrent UTIs if they had two separate culture-proven episodes of acute bacterial cystitis or pyelonephritis and associated symptoms within 6 months or three episodes within 1 year. All-cause 30 day mortality was determined by review of encounters in the EMR; survival was assumed in the absence of documentation of future encounters.

Symptom resolution times were calculated based on documentation in provider notes in the EMR. In the event that symptoms persisted throughout the patient's admission, time of resolution was documented as the time of discharge. Detection of ESBL and MIC data for piperacillin/tazobactam and carbapenems were collected as determined by the MicroScan WalkAway (Beckman Coulter, Brea, CA, USA) microbiology system.

### Statistical analysis

Outcomes were compared using a Mann–Whitney *U*-test, chi-square test or Fisher's exact test when appropriate. A *P* value less than 0.05 represented statistical significance. Analyses were performed using SPSS statistical program version 28.0 (SPSS, Inc., Chicago, IL, USA). Propensity score matching was conducted in R version 4.2.2 (R Core Team, 2022) to estimate the treatment effects of carbapenems and piperacillin/tazobactam on clinical outcomes. The propensity score was estimated via optimal matching using logistic regression with treatment arm (carbapenem or piperacillin/tazobactam) as the exposure and measures of clinical success as the outcome. The covariates used to generate the propensity scores included age, sex, ICU admission, Charlson Comorbidity Index (CCI) score, UTI classification (complicated, uncomplicated, pyelonephritis or catheter-related), number of urinary pathogens, presence of bacteraemia and presence of symptoms.

### Outcomes

The primary outcome was to compare rates of clinical success when using piperacillin/tazobactam with those when using carbapenems for the empirical treatment of UTIs caused by ESBL-producing organisms. Clinical success was defined as resolution of temperature within 48 h, resolution of symptoms or leukocytosis in the absence of symptoms within 48 h, and the absence of readmission with a UTI caused by an ESBL-producing organism within 6 months of discharge. Secondary outcomes included clinical success based on UTI classification, ICU admission, patients with concomitant ESBL bacteraemia, and history of recurrent UTIs. Other secondary outcomes included hospital length of stay (LOS), ICU LOS, time to clinical resolution (temperature and symptom resolution), and all-cause in-hospital and 30 day mortality.

### Ethics

The University of Tennessee Health Science Center Institutional Review Board approved this study for exempt review (21-08503-XM). No informed patient consent was required for purposes of this study due to its retrospective nature.

## Results

### Description of cohort

**Figure 1.**
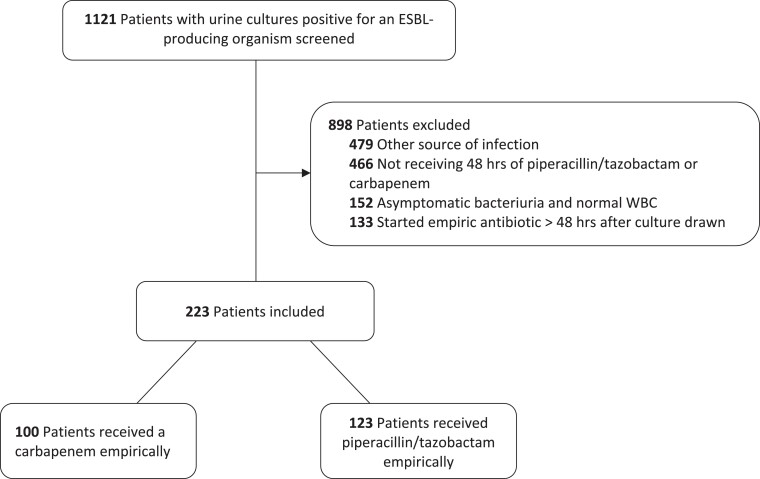
Flow diagram through study design.

A total of 1121 patients were screened, of which 898 were excluded. The most common reasons for exclusion were patients having another concomitant source of infection and not receiving at least 48 h of an empirical carbapenem or piperacillin/tazobactam. No isolates were resistant to piperacillin/tazobactam or a carbapenem among patients screened. Ultimately, 223 patients were included in the full cohort, with 100 (45%) patients receiving a carbapenem and 123 (55%) patients receiving piperacillin/tazobactam (Figure 1). Overall, 164 (74%) patients had symptoms documented at the time of empirical antibiotic initiation, and the most common urinary pathogen was *Escherichia coli* (*n* = 173, 78%) (Table 1). Baseline characteristics, including severity of illness (APACHE II) and comorbidity history (CCI score), were similar between both groups.

**Table 1.** Baseline characteristics^a^

	Full cohort			Matched cohort		
Characteristic	Carbapenem (*n* = 100)	TZP (*n* = 123)	*P* value	Carbapenem (*n* = 100)	TZP (*n* = 100)	*P* value
Female	69 (69)	75 (61)	0.21	69 (69)	67 (67)	0.76
Race						
** **Caucasian	49 (49)	66 (54)	0.49	49 (49)	60 (60)	0.12
** **African American	47 (47)	48 (39)	0.23	47 (47)	33 (33)	0.04
** **Other	4 (4)	9 (7.3)	0.29	4 (4)	7 (7)	0.35
Age (years), median (IQR)	71 (61, 81)	68 (57.5, 81)	0.52	71 (61, 81)	67.5 (56.8, 82)	0.58
Urological abnormalities^b^	37 (37)	37 (30)	0.28	37 (37)	28 (28)	0.17
Immunocompromised^c^	12 (12)	20 (16)	0.37	12 (12)	19 (19)	0.17
Concomitant ESBL bacteraemia	27 (27)	28 (23)	0.47	27 (27)	24 (24)	0.63
Symptoms present	71 (71)	93 (76)	0.44	71 (71)	74 (74)	0.63
** **Dysuria	15 (15)	13 (11)	0.32	15 (15)	12 (12)	0.53
** **Polyuria/urgency	6 (6)	7 (5.7)	0.92	6 (6)	6 (6)	1
** **Haematuria	12 (12)	15 (12)	0.96	12 (12)	12 (12)	1
** **CVA/flank pain	12 (12)	20 (16)	0.35	12 (12)	14 (14)	0.65
** **Suprapubic/abdominal pain	36 (36)	53 (43)	0.28	36 (36)	47 (47)	0.11
** **Hypotension	26 (26)	35 (28)	0.68	26 (26)	21 (21)	0.40
Severity of illness						
** **ICU admission	14 (14)	31 (25)	0.04	14 (14)	16 (16)	0.69
** **CCI, median (IQR)	2 (1, 4)	3 (1, 4)	0.62	2 (1, 4)	2.5 (1, 4)	0.66
** **APACHE II, median (IQR)	28.5 (20.2, 32)	23 (17.5, 28.5)	0.19	28.5 (20.2, 32)	21.5 (16.5, 25)	0.11
UTI classification^d^						
** **Uncomplicated cystitis	15 (15)	15 (12)	0.54	15 (15)	14 (14)	0.84
** **Complicated cystitis	61 (61)	73 (59)	0.80	61 (61)	58 (58)	0.67
** **Pyelonephritis	24 (24)	35 (28)	0.45	24 (24)	28 (28)	0.52
** **Catheter-related	23 (23)	22 (18)	0.34	23 (23)	20 (20)	0.61
Urinary pathogen^e^						
** ** *E. coli*	81 (81)	92 (75)	0.27	81 (81)	77 (77)	0.49
** ** *Klebsiella pneumoniae*	16 (16)	27 (22)	0.26	16 (16)	22 (22)	0.28
** ** *Proteus mirabilis*	6 (6)	7 (5.7)	0.92	6 (6)	4 (4)	0.52

a Characteristics reported as *n* (%) unless otherwise stated. CCI, Charlson Comorbidity Index; CVA, costovertebral angle; TZP, piperacillin/tazobactam; UTI, urinary tract infection.

b Urological abnormalities included nephrolithiasis confirmed with imaging, ureteric stents or nephrostomy tubes, prostatitis, renal abscess/cysts and neurogenic bladder.

c Immunocompromised patients included those receiving cytotoxic chemotherapy, immunosuppressants including >20 mg prednisone or equivalent per day for 28 days or more, or immunomodulatory biological agents; receipt of chimeric antigen receptor T-cell therapy or haematopoietic stem cell transplant within the previous 2 years; untreated or advanced human immunodeficiency virus with CD4 <200 cells/mL.

d Patients with catheter-related infections were also counted in one other classification.

e Patients included could have up to two organisms isolated.

**Figure 2.**
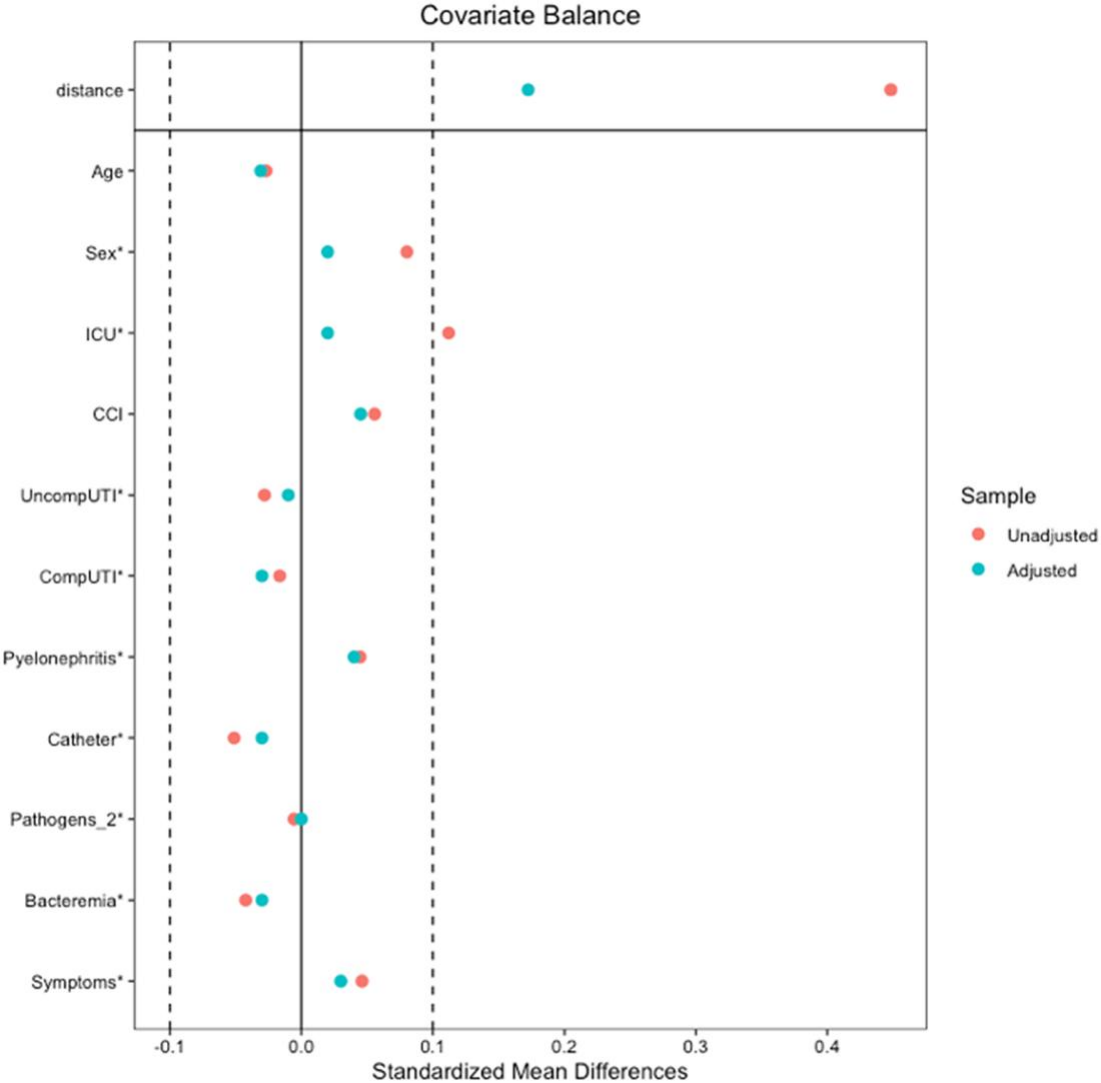
Standardized mean differences. Illustrates differences seen between groups for each listed data point before propensity score matching (unadjusted) and after (adjusted). After propensity score matching, all listed data points were similar between groups.

The variables of age, sex, ICU admission, CCI score, UTI classification, number of urinary pathogens, presence of bacteraemia and presence of symptoms were used to develop the propensity score model (Figure 2). APACHE II was not included as a variable for propensity score matching due to missing values for those not admitted to the ICU; therefore, ICU admission was used as a surrogate for severity of illness. A propensity score-matched subgroup analysis of 200 patients was performed, which included all 100 patients receiving a carbapenem and 100 of the original 123 patients receiving piperacillin/tazobactam. There were no significant differences between groups in the matched cohort. Distributions of UTI classifications were similar between groups: 61% versus 58% had complicated cystitis (*P* = 0.67), 15% versus 14% had uncomplicated cystitis (*P* = 0.84), and 24% versus 28% had pyelonephritis (*P* = 0.52) in the carbapenem and piperacillin/tazobactam groups, respectively (Table 1).

### Antibiotic treatment regimens

The most common antibiotic received within 48 h prior to a carbapenem or piperacillin/tazobactam in the full cohort was ceftriaxone in 38 (38%) patients in the carbapenem group and 32 (26%) patients in the piperacillin/tazobactam group (*P* = 0.06). The majority of patients (*n* = 99, 99%) in the carbapenem group received meropenem. Change in antibiotics after 48 h of receiving the study drug occurred in 65 (53%) patients receiving piperacillin/tazobactam and 33 (33%) patients receiving a carbapenem (*P* = 0.003). Of the patients whose empirical piperacillin/tazobactam was changed to another antibiotic, 52/65 (80%) subsequently received a carbapenem, and 26/33 (79%) of patients whose empirical carbapenem was changed were switched to ertapenem (Table 2). Although patients receiving piperacillin/tazobactam were more likely to switch to another antibiotic after 48 h, provider preference was the primary reason (66%) for changes to any other antibiotic. Provider preference was assumed if an antibiotic switch was made without mention of clinical worsening or alternative explanation in the medical chart. However, there was no significant difference in achieving the primary outcome of clinical success for patients receiving piperacillin/tazobactam alone compared with those switching from piperacillin/tazobactam to another antibiotic after 48 h (62.1% versus 53.8%, *P* = 0.36), respectively.

**Table 2.** Antibiotic regimens^a^

	Carbapenem (*n* = 100); *n* (%)	TZP (*n* = 123); *n* (%)	*P* value
Antibiotics initiated within 48 h of urine culture			
Ceftriaxone	38 (38)	32 (26)	0.06
Cefepime	6 (6)	1 (0.8)	0.05
Cefazolin	1 (1)	1 (0.8)	1.00
Levofloxacin	2 (2)	5 (4)	0.70
Ciprofloxacin	1 (1)	1 (0.8)	1.00
Fosfomycin	1 (1)	0 (0)	0.45
Cefuroxime	1 (1)	0 (0)	0.45
Gentamicin	0 (0)	1 (0.8)	1.00
Meropenem	99 (99)	0 (0)	n/a
Ertapenem	1 (1)	0 (0)	n/a
Piperacillin/tazobactam	0 (0)	123 (100)	n/a
Antibiotics switched to after receiving 48 h of study drug			
Meropenem	1 (1)	28 (23)	< 0.001
Ertapenem	26 (26)	24 (20)	0.25
Levofloxacin	1 (1)	5 (4)	0.24
Ciprofloxacin	1 (1)	3 (2)	0.63
Fosfomycin	2 (2)	0 (0)	0.20
SXT	1 (1)	3 (2)	0.63
Ceftriaxone	0 (0)	2 (2)	0.50
Ampicillin/sulbactam	1 (1)	0 (0)	0.45
Duration of study drug (days), median (IQR)	4.6 (3.2, 6.9)	3.2 (2.6, 6.9)	< 0.001

a Reported as *n* (%) unless otherwise stated. n/a, not applicable; SXT, trimethoprim/sulfamethoxazole; TZP, piperacillin/tazobactam.

The most commonly ordered empirical dose of piperacillin/tazobactam was 3.375 g (57%) with a frequency of every 6 h (87%). Meropenem was most frequently dosed at 1 g (64%) every 12 h (48%). Duration of therapy on the empirical study drug was significantly longer for patients receiving a carbapenem, with a median (IQR) of 4.6 days (3.2, 6.9 days) compared with 3.2 days (2.6, 6.9 days) for patients receiving piperacillin/tazobactam (*P* < 0.001).

### Matched cohort outcomes

**Figure 3.**
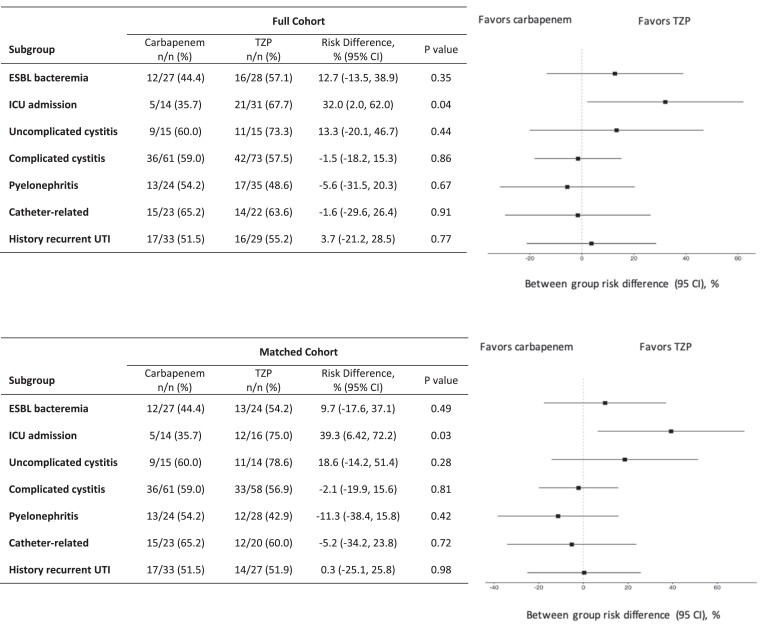
Subgroup analysis of clinical success^a^. TZP, piperacillin/tazobactam. ^a^Clinical success was defined as resolution of temperature within 48 h, resolution of symptoms or leukocytosis in the absence of symptoms within 48 h, and the absence of readmission with a UTI caused by an ESBL-producing organism within 6 months of discharge.

There was no significant difference in rate of clinical success between the two propensity score-matched groups for the empirical treatment of ESBL UTIs with piperacillin/tazobactam compared with a carbapenem, which was achieved in 58 (58%) of patients in the carbapenem group and 56 (56%) in the piperacillin/tazobactam group (risk difference −2.0%, 95% CI −15.7% to 11.7%, *P* = 0.76) (Table 3). There was also no significant difference in clinical success for predefined subgroups including patients with ESBL bacteraemia, different UTI classifications or a history of recurrent UTIs. However, patients admitted to the ICU achieved significantly higher rates of clinical success when receiving empirical piperacillin/tazobactam compared with a carbapenem, which was retained after propensity score matching (risk difference 39.3%, 95% CI 6.4% to 72.2%) (Figure 3). There was no significant difference in the secondary outcomes of median (IQR) time to clinical resolution [38.9 h (21.5, 50.9 h) versus 40.3 h (27.4, 57.5 h), *P* = 0.37] and ICU LOS [2.2 (1.3, 3.0) versus 1.8 (1.5, 2.9) days, *P* = 0.40] between the carbapenem and piperacillin/tazobactam groups, respectively (Table 4). Additionally, there were no significant differences with regards to in-hospital all-cause mortality (3% versus 3%, *P* = 1.00) or 30 day all-cause mortality (4% versus 2%, *P* = 0.68) between the carbapenem and piperacillin/tazobactam groups, respectively. However, patients receiving empirical piperacillin/tazobactam had a median (IQR) hospital LOS of 6.4 (4.3, 8.4) days compared with 7.0 (5.1, 10.6) days for those receiving a carbapenem, which was significantly shorter (*P* = 0.049).

**Table 3.** Clinical success outcomes

	Full cohort				Propensity score-weighted cohort			
Outcome	Carbapenem (*n* = 100) *n* (%)	TZP (*n* = 123) *n* (%)	Risk difference, % (95% CI)	*P* value	Carbapenem (*n* = 100) *n* (%)	TZP (*n* = 100) *n* (%)	Risk difference, % (95% CI)	*P* value
Primary outcome of clinical success^a^	58 (58)	70 (57)	−1.1 (−14.1, 12.0)	0.87	58 (58)	56 (56)	−2.0 (−15.7, 11.7)	0.78
Individual components of clinical success								
** **Symptom or WBC resolution in 48** **h	75 (75)	85 (69)	−5.9 (−17.7, 5.9)	0.33	75 (75)	67 (67)	−8.0 (−20.5, 4.5)	0.21
** **Temperature resolution in 48** **h	88 (88)	120 (98)	9.7 (2.6, 16.5)	0.005	88 (88)	97 (97)	9.0 (1.8, 16.2)	0.016
** **Absence of 6** **month readmission for ESBL UTI	83 (83)	100 (81)	−1.7 (−11.8, 8.4)	0.74	83 (83)	82 (82)	−1.0 (−11.5, 9.5)	0.85

TZP, piperacillin/tazobactam; UTI, urinary tract infection.

a Clinical success was defined as resolution of temperature within 48 h, resolution of symptoms or leukocytosis in the absence of symptoms within 48 h, and the absence of readmission with a UTI caused by an ESBL-producing organism within 6 months of discharge.

**Table 4.** Secondary outcomes

Outcome	Full cohort			Matched cohort		
	Carbapenem (*n* = 100)	TZP (*n* = 123)	*P* value	Carbapenem (*n* = 100)	TZP (*n* = 100)	*P* value
Time to clinical resolution (h), median (IQR)^a^	38.9 (21.5, 50.9)	40.3 (24.6, 56.6)	0.53	38.9 (21.5, 50.9)	40.3 (27.4, 57.5)	0.37
Hospital LOS (days), median (IQR)	7.0 (5.1, 10.6)	6.8 (4.8, 9.2)	0.12	7.0 (5.1, 10.6)	6.4 (4.3, 8.4)	0.049
ICU LOS (days), median (IQR)	2.2 (1.3, 3.0)	1.8 (1.1, 3.0)	0.52	2.2 (1.3, 3.0)	1.8 (1.5, 2.9)	0.44
In-hospital all-cause mortality, *n* (%)	3 (3)	3 (2.4)	1.00	3 (3)	3 (3)	1.00
30 day all-cause mortality, *n* (%)	4 (4)	2 (1.6)	0.41	4 (4)	2 (2)	0.68

LOS, length of stay; TZP, piperacillin/tazobactam.

a Clinical resolution was defined as resolution of temperature and symptoms.

## Discussion

There is growing interest in the definitive use of β-lactam/β-lactamase inhibitors (BL/BLIs) as carbapenem-sparing antibiotic options for patients with ESBL infections. A recent meta-analysis evaluating the efficacy of BL/BLIs versus carbapenems for UTIs caused by ESBL-producing Enterobacterales concluded that BL/BLIs are non-inferior to carbapenems in clinical and microbiological success and mortality.^11^ However, this review focused on newer generation BL/BLIs, such as ceftazidime/avibactam and ceftolozane/tazobactam, and their widespread use is limited by accessibility, cost and reservation for MDR organisms, including carbapenem-resistant Enterobacterales and *Pseudomonas*.^12,13^

Piperacillin/tazobactam has previously been studied in comparison with carbapenems for the definitive treatment of UTIs caused by ESBL-producing Enterobacterales. The only randomized trial evaluated 66 patients with ESBL *E. coli* UTIs and found no significant difference in clinical response between piperacillin/tazobactam and ertapenem.^14^ Although this study evaluated symptomatic resolution as a component of the primary outcome, it was limited by a small sample size and exclusion of patients with complicating urinary factors and urinary catheters, which are independent risk factors for acquiring a new ESBL UTI.^15^ Two small observational studies found no difference in outcomes between definitive therapy with piperacillin/tazobactam versus carbapenems, but these findings were limited by utilizing non-clinical measures of assessing efficacy, such as urinary microbiological eradication, change in antibiotics or rates of readmission without evaluating symptomatic outcomes.^5,6^ The only observational study restricting inclusion criteria to patients with symptoms showed no difference in the secondary outcome of symptom resolution at day 7, but findings limited the ability to directly compare the efficacy of piperacillin/tazobactam with carbapenems because most UTI symptoms, particularly for uncomplicated cystitis, may resolve spontaneously by that time.^7^ Lastly, the largest observational study (*N* = 492) showed no difference in the primary outcome of hospital LOS or in rates of microbiological eradication in patients with ESBL UTIs definitively treated with carbapenems versus non-carbapenem β-lactams.^8^ However, some significant limitations included the lack of assessment for symptoms upon inclusion and the assumption of clinical response for patients without documentation of improvement, risking inclusion of patients with asymptomatic bacteriuria who may not require treatment.

We sought to control for the limitations noted in previous definitive therapy studies by using a stringent primary outcome of clinical success, which included three aspects, one of which was resolution of either UTI symptoms (74% of patients overall) or systemic symptoms characterized by leukocytosis. Even utilizing this strict clinical assessment, we found no difference in clinical success between the piperacillin/tazobactam and carbapenem groups, including subgroup analyses of patients with complicated UTIs, pyelonephritis and catheter-related infections. Using these measures, we were able to limit inclusion of patients with asymptomatic bacteriuria. Additionally, this study uniquely evaluated time to clinical resolution, which provides clinically useful information regarding the differences in efficacy of both treatment options before antibiotic de-escalation, change in therapy or discharge.

Furthermore, unlike previous studies evaluating the use of piperacillin/tazobactam for ESBL UTIs, our study included patients with structural and functional urinary abnormalities and histories of recurrent UTIs, which more accurately reflects patients with risk factors for ESBL infections.^16^ Additionally, we particularly assessed outcomes related to the empirical use of these agents initiated within 48 h of urine culture collection and within 48 h of antibiotic therapy to limit confounders of delay in therapy and exposure to other treatments, respectively. Despite the common practice of switching to a carbapenem once an ESBL is identified, outcomes of this study support the efficacy of administering piperacillin/tazobactam empirically. The efficacy of piperacillin/tazobactam for urinary sources of infection may be explained by a lower bacterial burden compared with more severe infections such as bacteraemia or pneumonia, or by the pharmacokinetics of piperacillin/tazobactam in being able to achieve higher MICs to treat UTIs due to accumulation and elimination of drug in the urine.^17,18^ These findings showing no difference in clinical outcomes in patients who receive empirical piperacillin/tazobactam for ESBL UTIs are significant in that rising rates of ESBL UTIs in both hospital and community settings may trigger providers to empirically use more carbapenems. This, in turn, increases patients’ risk of developing *Clostridioides difficile* infection as well as a subsequent infection with a carbapenem-resistant organism.^19,20^ However, the results of this study may give providers pause in widely adopting empirical use of carbapenems when a patient has risk factors for an ESBL UTI given that no patients screened had urinary ESBL isolates resistant to piperacillin/tazobactam.

There are, however, limitations to the current study. First, the retrospective nature of the study allows for the risk of outcomes being affected by confounding variables. Although patients were matched for potential confounding variables, there were variables not included such as other antibiotics received before the study drug. Second, this is an observational study, which relies on accurate documentation in the EMR to assess improvement of subjective outcomes. Time to symptom resolution primarily relied on the documentation time of progress notes, which could have underestimated rates of clinical success depending on whether providers were able to enter notes describing symptom resolution within the first 48 h. However, this reflects real-world clinical practice, and the limitation was consistent between both piperacillin/tazobactam and carbapenem groups. Third, the rate of clinical success in the carbapenem group (58%) was lower than previously reported in the literature.^6–8,14^ The lower rate of clinical success is likely due to the requirement of having to meet all components of the primary outcome including urinary or systemic symptom and temperature resolution within 48 h along with the absence of readmission for an ESBL UTI within 6 months. Additionally, the 48 h time frame is more stringent than previously used durations for assessing symptom resolution, which may have contributed to the overall lower rates of clinical success observed. However, it was important to evaluate patients within the first 48 h in order to assess clinical outcomes based on empirical antibiotic choice prior to when identification and susceptibility results would be expected for urine cultures. This also means that failing to meet the primary outcome assessed at 48 h does not equate to clinical failure, which we tried to control for by evaluating time to symptomatic improvement, hospital LOS, and all-cause in-hospital and 30 day mortality between both groups. Lastly, due to a lack of access, we were unable to collect genotype data regarding the type of β-lactamase enzymes isolated; however, this is also reflective of real-world practice in that genotypic assessment is typically limited to large academic medical centres. We sought to control for this limitation by excluding patients whose identified pathogen was resistant to the study drug. Additionally, CTX-M accounts for the most prevalent ESBLs in the USA, which is inhibited by tazobactam and may contribute to comparable rates of clinical success in this study.^21^ Thus, application of these results should be used with caution in countries with other more prevalent causative ESBLs that are less likely to be inhibited by tazobactam.

### Conclusion

In conclusion, even when using the most stringent definition of clinical success that has been studied to date, we found no difference in outcomes with the empirical use of piperacillin/tazobactam compared with carbapenems for the treatment of ESBL UTIs, including for complicated cystitis and pyelonephritis. Based on the results of this study, clinicians could consider initiating piperacillin/tazobactam in patients with suspected ESBL UTIs as a carbapenem-sparing option; however, prospective and adequately powered studies are required to confirm these findings.
